# Rethinking Traditional Japanese Acupuncture and Moxibustion: Spread the *Hokushin-kai* Style of Acupuncture to the World

**DOI:** 10.1089/acu.2023.0134

**Published:** 2024-04-11

**Authors:** Takuya Masuda, Yu Takeshita, Yuichi Okumura, Shinpu Fujimoto, Renpu Fujimoto

**Affiliations:** ^1^ Division of General Internal Medicine & Rheumatology, Mitsui Memorial Hospital, Tokyo, Japan.; ^2^ Department of Traditional Medicine, Faculty of Medicine, Toho University Omori Medical Center, Tokyo, Japan.; ^3^ Hokushin-kai; Academic Society of Traditional Japanese Acupuncture and Moxibustion, Osaka, Japan.; ^4^ Acupuncture Clinic, Seimei-in, Tokyo, Japan.; ^5^ Acupuncture Clinic, Okumura-ikkando, Osaka, Japan.; ^6^ Acupuncture Clinic, Fujimoto-gensyudo, Osaka, Japan.; ^7^ Acupuncture Clinic, Fujimoto-kansyoin, Osaka, Japan.

Numerous recent clinical trials have revealed the effects of acupuncture. However, almost all of these investigations were based on the use of Traditional Chinese Medicine (TCM) or Western medicine. In the United Kingdom, only 3% of the practitioners treat patients with Japanese therapeutic methods.^1^ Thus, the real value of traditional Japanese acupuncture and moxibustion remains unknown to the world. In this Guest Editorial, we introduce the *Hokushin-kai* style–acupuncture as one of the real values of traditional Japanese acupuncture and moxibustion. We also suggest that clinical trials are needed to evaluate this acupuncture method.

During the Meiji era (1868–1912 ad), the Japanese government attempted to abolish the practice of acupuncture in favor of Western medicine, despite acupuncture's excellent clinical effects. At that time, acupuncturists resisted the policy and conducted research to support the use of acupuncture versus Western medicine.^2^

During the Syowa era (1926–1989 ad), Yanagiya and colleagues proposed that Japanese acupuncture should be based on ancient classic texts, such as *The Yellow Emperor's Inner Classic* (the *Huangdi Neijing*),^3^ which describes the principles of acupuncture. Moreover, various methods of Japanese acupuncture based on ancient classic texts, such as meridian therapy (*keiraku-tiryo*–style), were established.

Renpu Fujimoto inherited (14th generation) the ancient Japanese methods of *Kampo*, acupuncture, and moxibustion medicine; in 1979, he established the *Hokushin-kai*–style.^4,5^

The characteristics of this acupuncture method are as follows.

- Using TCM terminology as the common language of Oriental Medicine
- Taking the detailed history of the patient: life history; worries; systematic review of symptoms; dietary or drinking habits; and checking urine, feces, hair, nails, menstruation (for female patients), secretions (e.g., sputum, perspiration, mucus), and sleep conditions
- Performing a detailed physical examination: tongue; pulse; skin; facial color; and abdominal, back, and limbs' acupoints
- Using only 1 or 2 needles, as these are sufficient for treatment
- Applying a unique technique for needle insertion (*tounyu-shinpo*); using flexible needle mechanics, returning from deflection to straight to avoid patient's pain during insertion
- Using a noninsertion needle (*kodai-shin*); a replica of an acupuncture needle discovered in the grave of *Liu Sheng*, an ancient Chinese emperor
- Using a technique (*Dashin*) that involves tapping a needle on the abdomen^6^; the modified *Mubun-ryu* style was established during the Muromachi era (1336–1573 ad)
- Understanding the great value of the ancient classics; researching ancient Japanese classics and techniques as well as those of TCM.

An example is the treatment of low-back pain.^7^ Renpu Fujimoto treated >1 million patients with this technique.

Surprisingly, only 1 needle is sufficient for treatment in each session while treating with filiform needles. To achieve this, detailed history taking and accurate physical examination of the patient, as well as thorough analysis of the diagnosis and pathophysiology from the perspective of Oriental medicine, are necessary. Training in acupoint examination, based on the *Yellow Emperor's Inner Classic*^3^ text, is required. The findings of acupoint examination are classified into 2 categories, namely, Deficiency or Excess. In the state of Deficiency, Coldness, flaccidity, and perspiration occur. In the state of Excess, Heat, tension, and tenderness appear. In treatment, it is important to tighten the Deficient acupoint with a tonifying method and to loosen the Excessive acupoint using a drainage method. After treatment, a physical examination should be performed again to obtain evidence indicative of a good outcome.

Moreover, clinical advice on selection of acupoints or needles has been derived from extensive clinical experience. Acupoints in the extremities or thicker needles are preferable for procedures for which a stronger effect is expected. If a patient is weak (e.g., Qi Deficiency), it is safer to use thinner needles, *kodai-shin*, *Dashin*, or abdominal acupoints. *Kodai-shin* or *Dashin* do not impose a burden on the mental and physical status of patients; thus, these techniques are preferred for treating patients in the terminal phase of disease, children, and patients with hyperalgesia.

Vigorous research, reimplementation of ancient Japanese classics and techniques of acupuncture, and those of TCM, have enabled 1-needle acupuncture. This approach has led to new, previously unreported, clinical effects. For instance, *Fuyo* (ST-19) is noted as a point that merely causes Qi to descend and harmonizes the Stomach. *Hikichi-ryu,* a method of acupuncture utilized in the Edo era (1603–1868 ad), regards *Fuyo* and the surrounding area as a Liver point. The effect of *Fuyo* of smoothing the Liver and regulating Qi was shown through extensive clinical experience. Thus, *Fuyo* is an appropriate acupoint choice for treating a Liver–Stomach Disharmony pattern.

In the last 40 years, *Hokushin-kai* style acupuncture has been used to treat various medically unexplained symptoms or incurable organ diseases. However, almost all of these clinical reports were published only in Japanese. Therefore, knowledge regarding this acupuncture method worldwide and even among Japanese doctors is limited.

Clinical trials are warranted to evaluate the effectiveness of this traditional Japanese acupuncture method in clinical practice and promote the method's use in patients who may benefit from this approach worldwide. We suggest that medical providers utilizing TCM also adopt the *Hokushin-kai* style. Notably, this method of acupuncture involves the same terminology and logical thinking associated with TCM. Appropriate evaluation of the physical findings and selection of acupoints are useful for the clinical reasoning of Oriental medical diagnosis (i.e., pattern identification) and enhancement of treatment.

We would like to acknowledge the advice from the members and staff of the Hokushin-kai; Academic Society of Traditional Japanese Acupuncture and Moxibustion, Osaka, Japan.
